# Anesthesia departments’ readiness for the COVID-19 pandemic; a nationwide cross-sectional study in Israel

**DOI:** 10.1186/s12871-020-01173-w

**Published:** 2020-10-13

**Authors:** Barak Cohen, Yuval Baar, Shai Fein, Idit Matot

**Affiliations:** 1 Division of Anesthesia, Intensive Care, and Pain Management, Tel-Aviv Medical Center, 6 Weizmann St., 6423906 Tel-Aviv, Israel; 2 Outcomes Research Consortium, Cleveland Clinic, Cleveland, OH USA; 3 Division of Anesthesia, Operating Rooms, Pain, and Critical Care, Samson Assuta Ashdod University Hospital, Ben-Gurion University of the Negev, Ashdod, Israel

**Keywords:** Cross-sectional, Anesthesia department, Crisis management, COVID-19, Israel

## Abstract

**Background:**

The Coronavirus infectious disease 2019 (COVID-19) brings anesthesiologists and intensive care physicians to the mainstay of clinical workload and healthcare managements’ focus. There are approximately 900 anesthesiologists in Israel, working in non-private hospitals. This nationwide cross-sectional study evaluated the readiness and involvement of anesthesia departments in Israel in management of the COVID-19 pandemic. The impact on anesthesiologists’ health, workload, and clinical practices were also evaluated.

**Methods:**

An online questionnaire was distributed to all of anesthesia department chairs in Israel on April 14th. Each response was identifiable on the hospital level only. Informed consent was waived since no patient data were collected.

**Results:**

Response rate was 100%. A decrease of at least 40% in operating-room activity was reported by two-thirds of the departments. Anesthesiologists are leading the treatment of COVID-19 patients in 19/28 (68%) Israeli hospitals. Israel Society of Anesthesiologists’ recommendations regarding intubation of COVID-19 patients were strictly followed (intubations performed by the most experienced available physician, by rapid-sequence induction utilizing video-laryngoscopy, while minimizing the number of people in the room - about 90% compliance for each). Anesthesiologists in most departments use standard personal protective equipment when caring for COVID-19 patients, including N95 masks, face shields, and water-proof gowns. Only one anesthesiologist across Israel was diagnosed with COVID-19 (unknown source of transmission). All department chairs reported emerging opportunities that advance the anesthesia profession: implementation of new technologies and improvement in caregivers’ clinical capabilities (68% each), purchase of new equipment (96%), and increase in research activity (36%).

**Conclusions:**

This nationwide cross-sectional study had a complete response rate and therefore well-represents the anesthesia practice in Israel. We found that Israeli anesthesia departments are generally highly involved in the health system efforts to cope with the COVID-19 pandemic. Anesthesia and airway management are performed in a remarkably comparable manner and with proper protection of caregivers. Ambulatory anesthesia activity has dramatically decreased, but many departments find opportunities for improvement even in these challenging times.

## Background

The current coronavirus infectious disease 2019 (COVID-19) pandemic brings many challenges to the healthcare system. Since COVID-19 is primarily a respiratory disease, it often leads to respiratory failure, prolonged dependency on mechanical ventilation, and need for high intensity medical care [1]. Anesthesiologists are considered experts in airway management, mechanical ventilation, and intensive care. Although the critical role anesthesiologists played in the COVID-19 crisis is well appreciated, different aspects of their engagements on a national level have not been reported to date.

There are approximately 900 anesthesiologists in Israel working in non-private hospitals, caring for a national population of about 9 million people. Most Israeli anesthesiologists are registered in the Israeli Society of Anesthesiologists (ISA). Despite early assumptions according to which Israel will face thousands of COVID-19 mechanically ventilated patients in mid-April, the rapid transmission of the disease was contained, in part due to governmental orders of isolation, curfew, and social distancing [2]. The peak of the first “wave” of the pandemic spread occurred in mid-April, but even at that point, in contrast to earlier predictions, less than 200 COVID-19 patients were severely ill, of which less than 140 were mechanically ventilated. Shortly after that peak, transmission rate decreased as well as the number of COVID-19 patients and the pandemic was considered under control, until the second wave started in July. Nevertheless, the healthcare system was still making extensive preparations to cope with the original predictions at that point. One consequence was the abrupt decrease in elective surgical activity and anesthesia providers’ availability. The magnitude of this decrease and its ramifications were not measured. Additionally, as in the rest of the world, once the COVID-19 pandemic expanded, an acute shortage in medical equipment, personal protective equipment (PPE), and other supplies was noticed [3, 4]. The ISA has published its recommendations for safe airway management of patients suspected to carry SARS-CoV-2, including the use of N95 masks and goggles / face shields, but the adherence to these recommendations in light of the national shortage of PPE remains unknown. Similarly, the magnitude of SARS-CoV-2 infection in anesthesia personnel is also unknown.

The present national cross-sectional study was thus set to evaluate various aspects of the COVID-19 pandemic in anesthesia departments, including anesthesiologists’ level of involvement in leadership and management, implementation of safety considerations designed to minimize COVID-19 infection and spread among anesthesia personnel, effects on anesthesiologists’ health and availability, as well as anesthesia departments’ activity. More so, the perspectives of departmental chairs on the opportunities arising from the COVID-19 pandemic were evaluated.

## Methods

This nationwide cross-sectional study was conducted by an online questionnaire distributed to all anesthesia department chairs in Israel (*N* = 28), on April 14th 2020. The questionnaire was only identifiable on a departmental level. Reminders were set to be sent 3 days after the initial distribution to non-responders. The Tel-Aviv Medical Center institutional review board considered this survey of healthcare providers exempt from ethical approval, and consent was assumed by response to the survey. Since only organizational and institutional policy issues were addressed in this quality assurance project, with no identifiable or private health information, ethics committee approval and informed consent were waived.

The questionnaire consisted of 23 multiple-choice questions, some of which had an additional free-text response option. No question was mandatory. The main topics addressed by the survey were anesthesiologists’ level of involvement in the COVID-19 crisis management, use of different components of PPE, modifications to the anesthesia induction and airway management methods, ramifications of the crisis on anesthesiologists’ health and availability, and perceived opportunities arising from the situation. A full copy of the survey is available as **Supplementary File** **1**.

Data were analyzed and presented with descriptive statistics. Pearson’s Chi-square test was used in a post-hoc sensitivity analysis to compare the responses between large (number of hospital beds > 500) and small (< 500 hospital beds) anesthesia departments. We used a convenience sample of all anesthesia departments in Israel. This manuscript adheres to the applicable STROBE guidelines for reporting of cross-sectional studies.

## Results

The questionnaire was completed and submitted by all 28 anesthesia department representatives within 2 days, so no reminders were sent (response rate 100%). Average (standard deviation) completion time was 10 (7) minutes. One third (9/28, 32%) of anesthesia departments include the intensive care unit (ICU), and one half (14/28, 50%) are in large hospitals (> 500 beds).

*Leadership role and responsibilities:* In two thirds of hospitals (19/28, 68%), anesthesiologists were leading COVID-19 patients’ treatment, either by themselves or in collaboration with intensive care and internal medicine physicians. There was no difference between hospitals in which the ICU is part of the anesthesia department (6/9, 67%) and those in which they are managed independently (13/19, 68%). Other responsibilities of anesthesia departments included managing COVID-19 patients’ airway (e.g., dedicated rapid response teams, 25/28), taking part in hospital crisis management and leadership (24/28), supervising the operating rooms (24/28), and clinical training and simulation to other disciplines in preparation to COVID-19 patients’ treatment (19/28).

*Anesthesia activity*: As for elective surgical volume and anesthesia services, two thirds of departments reported a decrease of at least 40% in operating room activity (19/28, 68%), and 75% of departments reported cancellation of non-operating-room anesthesia cases.

**Fig. 1:**
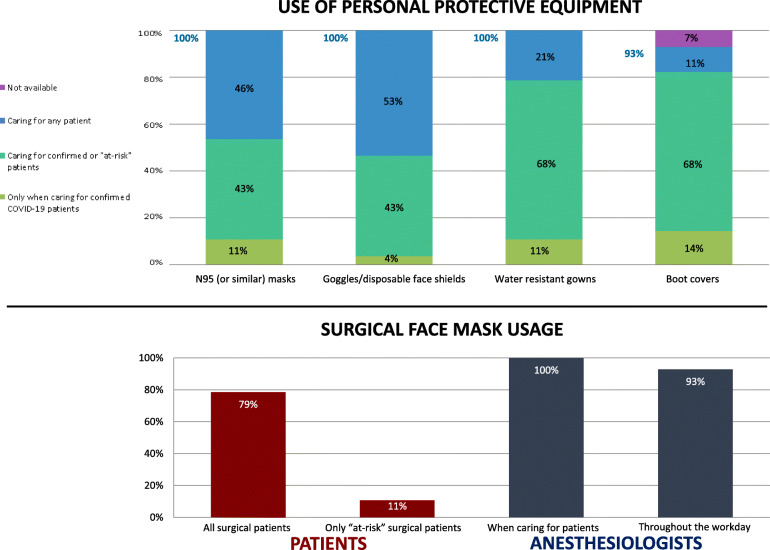
Use of personal protective equipment. Upper panel – percentage of anesthesia departments using different components of protective equipment. Lower panel – usage of surgical face masks by patients and caregivers

*Safety considerations* (Fig. 1): Nearly all departments reported that anesthesiologists are wearing a surgical face mask constantly throughout the working day within the hospital (26/28, 93%), and in 79% of hospitals all patients arriving to the operating room also wear one. An aerosol protective mask (N95 or similar) is used in all hospitals when caring for confirmed COVID-19 patients, and in half of the departments when treating any patient arriving to the operating room (13/28, 46%). In most hospitals, one N95 mask is used throughout the day, and not replaced between cases (“re-use”, 18/28, 64%). Goggles or face shields are used by all departments, either in all cases (15/28, 54%), or when caring for patients at-risk for SARS-CoV-2 infection. Similarly, water resistant gowns were used in all departments, either for all cases (6/28), for “at risk” cases (19/28), or only for confirmed COVID-19 cases (3/28). Boot covers were also commonly used (not available in only 2/28 centers). Ten departments reported using plastic covers on patients’ heads to minimize potential aerosol exposure, and seven reported the use of aerosol boxes. Specific training for donning and doffing of PPE was performed in 86% of departments (24/28).

Two thirds of departments reported working in segregated shifts in order to minimize exposure and risk of transmission across caregivers. All but one hospital reported to have a dedicated operating room for COVID-19 cases (27/28, 96%), usually with a separate access than the other operating rooms (16/27, 59%), and with a dedicated area for donning and doffing of PPE (19/27, 70%). Eleven hospitals noted this operating room operates under negative pressure.

*Airway management:* When asked about specific components of airway management in COVID-19 patients, vast majority of departments reported that these intubations are performed by the most experienced provider (26/28, 93%), using rapid sequence induction (25/28, 89%), and after leaving only the minimal number of people necessary in the room (26/28, 93%). Video-laryngoscopes were the default device utilized according to 93% of respondents (26/28), and use of disposable airway equipment was reported by 75% of departments (21/28).

*Anesthesia personnel considerations*: One quarter of departments reported that at least 10% of their manpower was absent due to home isolation orders for suspected contact with a COVID-19 positive person at some point during the current crisis (7/28, 25%). Nevertheless, only one anesthesiologist throughout the country was reportedly sick with COVID-19, while the source of infection remains unknown.

*Prospects arising from COVID-19 crisis* (Table 1): Organizational processes related to anesthesia activity that were promoted by the pandemic as noted by anesthesia chairs included purchase of new required equipment (such as video-laryngoscopes and ultrasound machines), improvement in clinical care and training of caregivers, implementation of new technologies, execution of tele-medicine practices, and the launch of new research activities.

**Table 1:** perceived opportunities arising from the COVID19 crisis among Anesthesia department chairs in Israel (*n* = 29)

Perceived opportunities	Prevalence, %
Purchase of new equipment	96%
Training and improved clinical capabilities of caregivers	68%
Technological innovations implemented	68%
Transition to tele-medicine	57%
Research	36%

A sensitivity analysis evaluating differences between large and small hospitals (cut-off 500 beds) did not find any significant differences.

## Discussion

This cross-sectional study collected data from all anesthesia departments in Israel, with a complete response rate. It was concluded within 2 days, and therefore well-represents the nationwide situation in the Israeli healthcare system in mid-April. Of note, hospital preparations for a large-scale pandemic aimed at coping with several thousands of mechanically ventilated COVID-19 patients were mostly completed at that point, while the actual number of ventilated patients across the country on April 16th was 140 [5].

The dramatic decrease in surgical and procedural volume requiring anesthesia services reported in all hospitals around the globe was also evident in Israel as noted in the present study. Two key factors contributed to this in Israel: First, national regulations that went into effect in mid-March mandated active decrease of elective medical and surgical activity in public hospitals [5]. Secondly, regulatory bodies mandated people to stay at home except for extreme circumstances, forbidding any kind of outdoor personal activity, thus decreasing the chance of acute injuries.

We found that anesthesia departments in Israel are highly engaged in COVID-19 crisis and in about two thirds of hospitals anesthesiologists led the way through the crisis. Anesthesiology chairs were the primary managers of different aspects of hospitals’ preparation processes, staff anesthesiologists trained other physicians in ventilation modes, conducted simulations, and many were shifted away from the operating room to treat patients in the ICUs.

The nature of their job puts health workers overall, and anesthesiologists in particular, at increased risk of being infected with COVID-19. During the SARS outbreak In Toronto, Canada in 2003, one-third to one-half of all infections were in healthcare workers [6, 7]. An early report from Wuhan county in China found that as many as 29% of hospitalized patients with COVID-19 were healthcare professionals, that acquired SARS-CoV-2 infection while working in the hospital [8]. More so, once the disease becomes prevalent among healthcare workers, the workload on their healthy colleagues clearly increases. For reference, on the day of the present study survey closure, over 1400 Israeli healthcare professionals were under home isolation due to suspected unprotected contact with a positive SARS-CoV-2 person.

Protecting anesthesia teams has thus been the focus of hospital administrations across the country. One initiative was to train the staff in donning and doffing of PPE. A retrospective study evaluating risk factors for healthcare workers’ acquisition of the original SARS infection in 2003 found that workers who did not undergo proper infection-control training had higher risk of transmission as a result of caring for SARS-positive patients [9]. Several national associations and international health organizations recommend specific training of PPE donning and doffing to facilitate healthcare workers’ safety [10–13], but reports of actual adherence to these recommendations are scarce. A recent survey among Turkish anesthesiologists found that only one third of respondents (37%) underwent specific COVID-19 training [14]. In contrast, a review of the COVID-19 response in a tertiary hospital in Singapore reported thorough PPE training of all 180 anesthesia physicians by a small group of staff anesthesiologists [15]. Similarly, in Israel, as evident in the present study, the majority of anesthesiologists underwent such training.

Access to protective equipment, during high risk procedures or while being exposed to suspected or infected COVID-19 patients, is another critical measure to avoid infection with COVID-19. The local, national, and international shortage of PPE has led the most influencing agencies as the World Health Organization and the United States Center for Disease Control and Prevention to publish guidelines on potential extended use and re-use of some disposable protective items [11, 13, 16, 17]. Most of the published data about hospitals’ and nations’ preparedness report proper use of PPE, although much has also been published about improvised solutions to reduce aerosol exposure, presumably in low-resource situations where appropriate PPE is in shortage [18–20]. In Israel, following the Israeli Society of Anesthesiologists’ recommendations, practically all anesthesia departments reported proper use of PPE when caring for confirmed or suspected COVID-19 patients, mostly in an extended use or re-use fashion. Many have also expanded the use of aerosol protection to all surgical cases.

Several national anesthesia societies have published recommendations on the proper modifications to anesthesia induction and airway management [10, 12, 21]. Aside of the use of aerosol-level PPE, these often include rapid-sequence inductions, use of video-laryngoscopes, leaving the minimal necessary number of people in the room, providing care by the most experienced provider, avoidance of mask ventilation and fiberoptic bronchoscopy unless specifically indicated, as well as avoidance of tubing disconnections. The Israeli Society of Anesthesiologists has published similar recommendations that were strictly followed with impressive compliance by Israeli anesthesiologists, presumably contributing to caregivers’ safety and setting an example to other disciplines. Unsurprisingly, some of these practices were extended to be used when caring for “at risk” patients, and sometimes to all surgical patients. These practices are neither supported nor addressed by most official guidelines, leaving a gap that was filled by local initiatives. Taken together, proper use of PPE, its availability to anesthesiologists despite national shortage, and adherence to Israeli Society of Anesthesiologists’ airway management regulations could, in part, explain the fact that out of about 900 anesthesiologists working in a non-private setting in Israel, only one was reported positive for SARS-CoV-2, the source of which remains unknown.

Finally, many departments were also able to find opportunities in the health crisis. Examples include innovative solutions to long-lasting gaps in information technologies, acquisition of long-needed medical equipment, and promotion of research projects.

The main limitation of our study originates from its design – an survey offering anesthesia department chairs to participate and report the situation, as well as perceived achievements and challenges, of the departments they manage. Although anonymous in nature, we cannot verify the objective accuracy of participants’ answers, and some degree of reporting and recall biases cannot be ruled-out.

## Conclusion

This cross-sectional study provides a glimpse on anesthesia departments’ engagement in the COVID-19 crisis in Israel. It shows the inspiring role the anesthesia leadership took upon itself while keeping its members protected and safe, and despite the calamity and uncertainty found prospects for upgrading the profession. This encouraging message raises an opportunity to establish a more proper acknowledgement of the anesthesia profession and its importance, which used to suffer poor reputation and to lack public understanding of the various roles anesthesiologists are able to take in different setups [22].

## Supplementary information

**Additional file 1.**

## Data Availability

Not applicable.

## Abbreviations

COVID-19: Coronavirus infectious disease 2019
ISA: Israeli society of anesthesiologists
PPE: Personal protective equipment
ICU: Intensive care unit
