# Supplementary material for: Anesthesia departments’ readiness for the COVID-19 pandemic; a nationwide cross-sectional study in Israel
Source: BMC Anesthesiol. 2020 Oct 13;20:262. doi: 10.1186/s12871-020-01173-w (PMC7552575; doi:10.1186/s12871-020-01173-w)
Supplement: Supplementary file 1 — Additional file 1. [file 12871_2020_1173_MOESM1_ESM.pdf]

Anesthesia and Anesthesiologists during the COVID-19 pandemic

This survey is conducted in order to better understand how hospitals and anesthesia departments in Israel are handling the COVID-19 pandemic.

1. Medical Center:

____

2. Is the intensive care unit part of the anesthesia department ? (Select one option)

- ◯ Yes
- ◯ No

3. Who is <u>leading</u> the management of COVID-19 patients? (Select one option)

- ◯ Anesthesiologists
- ◯ Intensivists
- ◯ Internal Medicine physicians
- ◯ Anesthesiologists and intensivists
- ◯ All above

4. What is the responsibility of the anesthesia department during the COVID19 crisis? (choose all that apply)

- ☐ Running the operating rooms
- ☐ Managing COVID19 patients' airway (when alarmed to do so – "crush teams")
- ☐ Treating COVID19 patients in dedicated units (intensive care or other)
- ☐ Administering simulations (mechanical ventilation/ intubation/ other) to other disciplines
- ☐ Writing treatment protocols
- ☐ Taking part in the hospital leadership for COVID19 crisis

5. Do you work in "capsules" – i.e in segregated teams (12/24 or other)? (Select one option)

- ◯ Yes
- ◯ No

6. Which fraction of the anesthesia department has been put in home quarantine since the beginning of the current crisis? (Select one option)

- ◯ 0%
- ◯ <10%
- ◯ 10 - 25%
- ◯ 25 - 50%
- ◯ >50%

7. Has any of the anesthesiologists in your department been infected with COVID-19? (Select one option)

- ◯ Yes
- ◯ No

NOTE : Answer the below question only if answer to Q#7 is Yes

8. How many?

(Select one option)

- ◯ 1
- ◯ 2
- ◯ 3
- ◯ 4
- ◯ 5
- ◯ >5

NOTE : Answer the below question only if answer to Q#7 is Yes

9. What is the source for this infection (check all relevant if several anesthesiologists were infected)?

- ☐ A patient they treated
- ☐ A colleague in the hospital
- ☐ Outside of hospital source
- ☐ Unknown

The following questions refer to types of personal protective equipment (types and indications) that are used in your department

10. A) Surgical face mask - SFM (check all that apply)

- ☐ Every patient coming for surgery is wearing one
- ☐ Only patients defined “at risk” coming for surgery are asked to wear one
- ☐ Anesthesiologists in the OR wear SFM when treating any patient
- ☐ Anesthesiologists wear SFM at all times (when in hospital)

11. B) N95 (or similar) masks are used in the OR when (check all that apply)

- ☐ Treating any patient
- ☐ when treating “at risk” patients
- ☐ when treating confirmed COVID19 patients

12. C) For how long is one N95 mask being used in the OR, when NOT treating confirmed COVID-19 patients?

(Select one option)

- ◯ One N95 mask per day
- ◯ One N95 mask per patient
- ◯ For multiple days
- ◯ Not available

13. D) Goggles/disposable face shields are used in OR when?

- ☐ Treating any patient
- ☐ when treating "at risk" patients
- ☐ when treating confirmed COVID19 patients
- ☐ Not available

14. E) A water resistant gown is used in the OR when? (check all that apply)

- ☐ Treating any patient
- ☐ when treating "at risk" patients
- ☐ when treating confirmed COVID-19 patients
- ☐ Not available

15. F) A boot cover is used in the OR when? (check all that apply)

- ☐ Treating any patient
- ☐ when treating "at risk" patients
- ☐ when treating confirmed COVID19 patients
- ☐ Not available

16. G) Other personal protective equipment available and used in the OR? (check all that apply)

- ☐ Aerosol box
- ☐ Plastic cover
- ☐ Another type of face cover or mask for intubators
- ☐ Other (Please specify) _______________

17. Your protocol for intubating patients with confirmed COVID-19 in the OR includes? (check all that apply)

- ☐ Minimize number of caregivers in the room
- ☐ The most experienced intubator performs the intubation
- ☐ Rapid sequence induction
- ☐ Use of videolaryngoscope
- ☐ Use of disposable airway equipment

18. Did all anesthesiologists undergo simulation / training in donning and doffing personal protective equipment ? (Select one option)

- ○ Yes
- ○ No

19. Is there a dedicated OR for suspected or confirmed COVID-19 patients in need of operation? (Select one option)

- ○ Yes
- ○ No

NOTE : Answer the below question only if answer to Q#19 is Yes

20. If yes, does it include (choose all relevant)

- ☐ Negative pressure environment
- ☐ Separate access (aside of other ORs)
- ☐ Dedicated area for doffing and donning

21. Has any of the following processes occurred as a result of the COVID-19 crisis?

- ☐ Transition to telemedicine
- ☐ Purchase of equipment
- ☐ Technological innovations implemented
- ☐ Training and improved clinical capabilities of caregivers
- ☐ Research
- ☐ Other (Please specify) _______________

22. Following the COVID-19 pandemic, OR activity has been reduced by approximately

(Select one option)

- ○ 0%
- ○ 10%
- ○ 20-25%
- ○ 30-40%
- ○ 40-50%
- ○ > 50%

23. In general, has most of the Non-operating room activity (NORA) been cancelled?

(Select one option)

- ○ Yes
- ○ No

Thank you for your valuable time!
